# Molecular diagnostic methods for rapid diagnosis of central nervous system infections

**DOI:** 10.3389/fmedt.2025.1497512

**Published:** 2025-04-22

**Authors:** Mallikarjuna Pedduru Venkatareddy, Dinesh Upadhya, Prakash Peralam Yegneswaran, Aneena Varghese, Suryadipali Pahadasingh, Arvind N. Prabhu, Kavitha Saravu, Kavitha S. Shettigar

**Affiliations:** ^1^ Department of Medical Laboratory Technology, Manipal College of Health Professions, Manipal Academy of Higher Education, Manipal, Karnataka, India; ^2^ Center for Molecular Neurosciences, Kasturba Medical College, Manipal, Manipal Academy of Higher Education, Manipal, Karnataka, India; ^3^ Department of Microbiology, Kasturba Medical College, Manipal, Manipal Academy of Higher Education, Manipal, Karnataka, India; ^4^ Department of Neurology, Kasturba Medical College, Manipal, Manipal Academy of Higher Education, Manipal, Karnataka, India; ^5^ Department of Infectious Diseases, Kasturba Medical College, Manipal, Manipal Academy of Higher Education, Manipal, Karnataka, India

**Keywords:** central nervous system infections, molecular diagnostics, polymerase chain reaction, real-time PCR, next-generation sequencing

## Abstract

Central nervous system infections (CNSI) are serious life-threatening conditions caused by bacteria, viruses, fungi, and parasites and lead to high morbidity and mortality worldwide. Therefore, rapid identification of causative organisms and appropriate treatment are important. The traditional identification methods are time-consuming and lack sensitivity and specificity. Although culture method is gold standard for CNSI, it is time-consuming and microbiology reporting requires several days. Multiplex PCR assays can detect multiple pathogens simultaneously in clinical samples and overcome the limitations of conventional identification techniques. Despite the availability of several commercial molecular-based platforms for the detection of pathogens causing CNSI, there are still limitations in terms of cost, false positive results, and false negative results, which are limited to targeted pathogens in the panel. Moreover, validation of many commercially available and in-house laboratory-developed molecular assays is still lacking. In addition, molecular diagnostic tests need to be used in correlation with the clinical context to ensure better diagnosis and management of infections.

## 1 Introduction

Central nervous system infections (CNSI) are potentially life-threatening conditions, leading to high mortality and morbidity worldwide. The pathogens that cause CNSI are bacteria, mycobacteria, fungi, viruses, and parasites. Therefore, rapid diagnosis, timely treatment, and appropriate management of patients are important (1–3). Although various bacterial species cause CNSI, the most common causative bacteria reported over the last five years in neonates and children are *Neisseria meningitidis, Haemophilus influenzae type b*, *Streptococcus pneumoniae*, *Streptococcus agalactiae*, and *Listeria monocytogenes*. Additionally, the prevalence of these causative agents depends on the age of the patient and geographical region (4–6). In India, the frequency of bacterial meningitis in children (0–14 years old) ranges from 0.5% to 61.8%, and in meningitis patients of all age groups (0–75 years), the frequency varies from 8.7% to 78.9% (7). According to reports from the Global Burden of Disease (GBD) study of 2019, CNSI is the sixth most common disease among children under the age of 10 years (8) with 2.82 million incident cases and 3,18,400 estimated deaths (9, 10). Over 1.2 million bacterial meningitis cases are estimated to occur annually globally (11). In addition, 1,35,000 cases of mortality due to bacterial meningitis have been reported. The mortality rate in high-resource settings ranges from 10% to 20%, whereas in lower-resource settings, the mortality rate is approximately 50% (6, 12). Viruses, including enteroviruses, herpesviruses, mumps, and flaviviruses with an estimated incidence between 0.26 and 17 cases per 1,00,000. The incidence of aseptic meningitis depends on the age or vaccination status of the population (6). Children and elderly people are at increased risk of aseptic meningitis. Enteroviruses are a common cause of acute inflammation involving the CNS (1, 2, 13). Tuberculous meningitis (TBM) is a severe form of extrapulmonary tuberculosis (EPTB), which constitutes approximately 1%–5% of new cases each year (14). Tuberculous meningitis accounts for a mortality rate of up to 50% in HIV-infected individuals (15). Cryptococcal meningitis is associated with approximately 15% of AIDS-mediated deaths worldwide. In India, the prevalence of cryptococcal meningitis ranges from 2.09% to 53.1% and is most prominent in patients with HIV/AIDS (7). CNSI caused by non-cryptococcus fungi typically results from fungal dissemination (6). The accurate clinical diagnosis of CNSI is clinically challenging because of the overlapping clinical features and symptoms (1, 16).

In recent years, molecular techniques such as nucleic acid amplification tests (NAATs) have been widely used to accurately diagnose bacterial and fungi mediated CNSI. NAATs provide higher pathogen detection rates with higher sensitivity and specificity than conventional methods do (17–19). The utility of real-time polymerase chain reaction (real-time PCR) for diagnosing infections has increased over the last few years (20). Currently, only one FDA-approved multiplex method is the Biofire FilmArray® ME Panel (BioFire, bioMerieux, Salt Lake City, USA), which detects up to 14 pathogens, including bacteria, viruses, and fungi, in cerebrospinal fluid (CSF) samples and is widely used (18). In addition, other methods, including a second closed qualitative multiplex PCR cassette-based panel, the *in vitro* Diagnostic Devices Directive (CE-IVDD)-marked device, and the QIAstat-Dx Meningitis/Encephalitis (ME) Panel (QIAGEN, Germany), can simultaneously identify and detect up to 15 pathogens (eight bacteria and six viruses and one fungus) in CSF samples. Both panels require only a 200 µl sample without the need for prior extraction, and the yield results are less than 2 h (21). In addition, a novel syndromic nucleic acid amplification-based evaluation system (Xcyton Diagnostics, India) was developed in India and is available to detect 22 pathogens simultaneously in CSF samples (22).

Several other FDA-approved rapid molecular assays are available to detect pathogens from CSF samples, including the Xpert EV assay (Cepheid) for enterovirus detection, the Simplexa HSV 1 & 2 Direct Kit (Focus Diagnostics), and the MultiCode RTx HSV 1 & 2 Kit (Luminex Corporation) to detect herpes viruses 1 and 2 (18). In addition, other commercial molecular panels are available, including multiplex-based Allplex Meningitis panels (SeeGene, Seoul, Republic of Korea), ePlex CNS panels (GenMark Dx, Carlsbad, CA, USA), Fast Track Diagnostics panels such as the FTlyo Viral Meningitis Panel, FTlyo Bacterial Meningitis, FTD Neuro 9, and FTD Neonatal Meningitis (Fast Track Diagnostics, Esch-sur-Alzette, Luxembourg). Furthermore, the Meningitis Viral 1 ELITe MGB® Panel (Elitech InGenious, Puteaux, France) and the Meningitis Viral 2 ELITe MGB® Panel (Elitech InGenious, Puteaux, France) are available to detect pathogens of CNSI. However, clinical validation of these tests is still lacking (23). This review discusses the benefits, limitations, and future scope of current molecular diagnostic methods for detecting pathogens of CNSI in CSF samples.

## 2 Methods

Relevant articles were identified through literature search on PubMed, Embase, Scopus, Ovid Medline, and Web of Science databases and were restricted to articles published between 2010 and 2023. The following keywords along with Boolean operators AND, OR and NOT, in addition to truncations were used to obtain relevant articles: “molecular diagnostics”, “CNS infection”, “meningitis”, “bacterial meningitis”, “cryptococcal meningitis”, “fungal meningitis”, “viral meningitis”, “aseptic meningitis”, “tuberculous meningitis”, “rapid diagnostics”. We have screened cohort studies, cross-sectional studies, narrative reviews, systematic reviews and case control studies. Only full text articles published in English were included. The title and abstract of the articles were screened and relevant articles were included for the review. The final dataset included 128 full-text articles, meeting the inclusion criteria. The identified articles were reviewed and then classified based on the study and were then collated to understand the various molecular diagnostic methods to detect pathogens of CNSI.

## 3 Current approaches and diagnostic limitations

**Figure 1:**
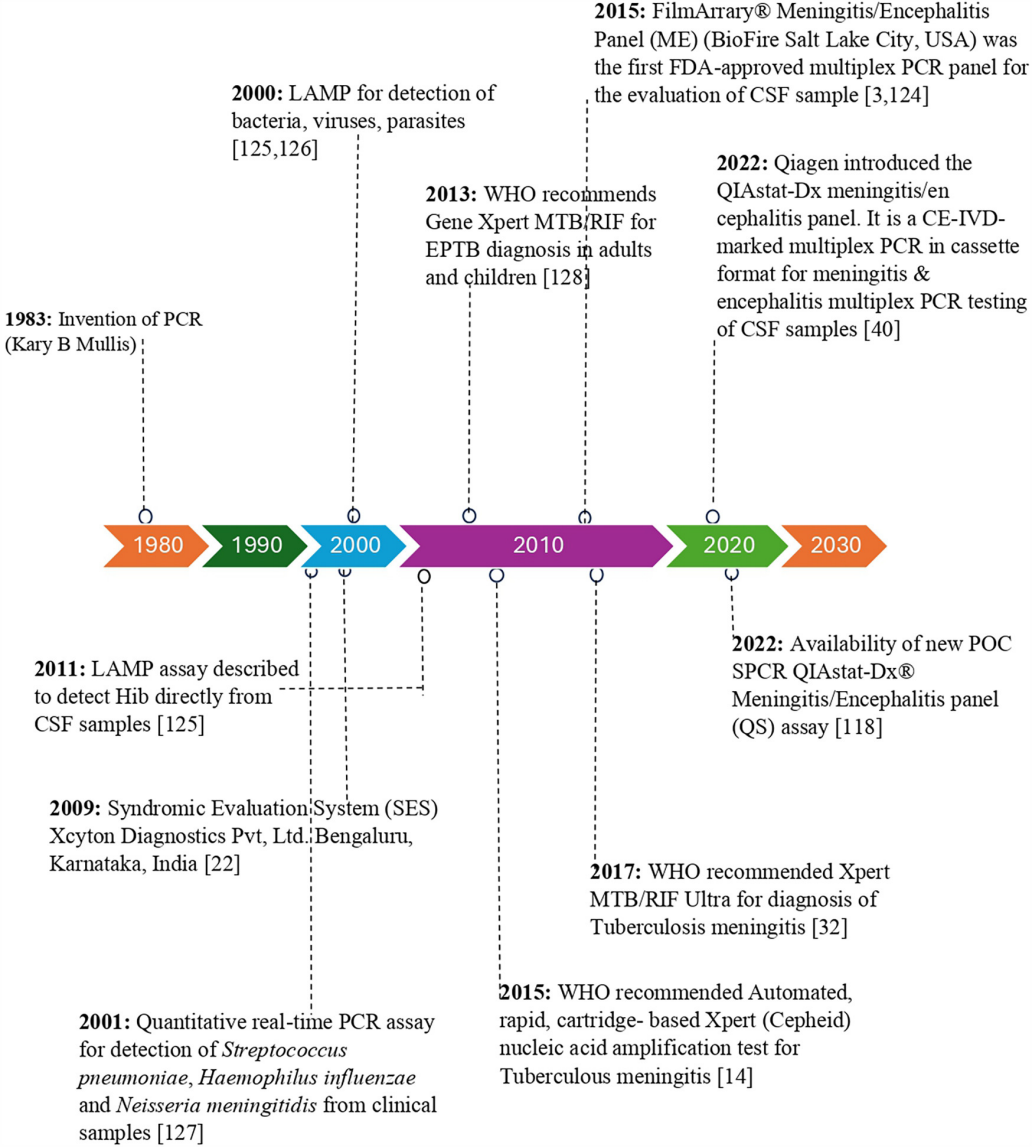
Timeline of commercial and investigational molecular diagnostic tests for diagnosis of meningitis/encephalitis etiology.

Traditionally, the diagnosis of CNSI is based on the analysis of cytological and biochemical profiles of CSF, such as protein, glucose, leukocyte count, Gram staining, fungal yeast detection, and acid-fast bacilli (AFB) staining, for tuberculous meningitis. The conventional culture method for CSF samples remains the gold standard for detecting bacterial meningitis, Cryptococcal meningitis, and tuberculous meningitis (6, 21, 24–26). However, the culture method is time-consuming, and the results are affected by prior antibiotic treatment and hence lack sensitivity (21). Currently, both conventional and molecular approaches are used to diagnose CNSI. CSF Gram stain examination can provide presumptive identification in 50% to 90% of cases. However, its sensitivity ranges from 60% to 90%. In addition, the sensitivity is reduced if the patient is receiving empirical antibiotic treatment before lumbar puncture. The sensitivity of CSF culture ranges between 70% to 90% (6, 27, 28). Encapsulated yeast detection via India ink staining of CSF samples for direct examination of Cryptococcus is possible; however, its sensitivity is as low as 42%. Although conventional fungal culture is the gold standard for diagnosing cryptococcal meningitis, culture requires 10 days for growth to occur (6). Ziehl–Neelsen staining of CSF for the detection of tubercle bacilli is used for the diagnosis of tuberculous meningitis; however, its sensitivity ranges from 10% to 60% (29). CSF culture remains the gold standard for the diagnosis of tuberculous meningitis; however, its sensitivity ranges from 15% to 66%, and its growth requires more than 2 weeks. CSF adenosine deaminase detection is a simple, rapid, and widely used standard method in lower-resource settings. A recent meta-analysis reported that adenosine deaminase has a sensitivity of 89% and specificity of 91% for diagnosing tuberculous meningitis (30, 31). CSF lactate has a sensitivity of 96% and a specificity of 100% in differentiating bacterial meningitis from aseptic meningitis. However, elevated levels of lactate are also observed in other infectious and noninfectious conditions. C-reactive protein and procalcitonin markers in CSF have sensitivities of 92% and 96.4% and specificities of 80% and 84.4%, respectively; however, these markers are not confirmatory markers of CNSI (6). Furthermore, GeneXpert MTB/RIF (Cepheid) is endorsed by the World Health Organization (WHO) and has a sensitivity ranging from 50% to 70% for diagnosing tuberculous meningitis (6, 32, 128). However, currently available molecular methods such as the line probe assay (LPA) and loop-mediated isothermal amplification (LAMP) are cost-prohibitive for low- and middle-income countries. Matrix-assisted laser desorption/ionization time-of-flight mass spectrometry (MALDI-TOF MS) is not commonly used for the diagnosis of CNSI in CSF samples. Furthermore, NAATs, 16S rRNA PCR, sequencing-based next-generation sequencing (NGS), and metagenomic next-generation sequencing (mNGS) methods are extremely expensive, require laboratory expertise and infrastructure, and their utility in the diagnosis of CNSI remains unclear (6, 33, 34). The emergence of various commercially available tests for the diagnosis of CNSI is represented in Figure 1. Viruses are detected in CSF samples using PCR, the viral culture method, and antibody-based detection of viral pathogens. PCR remains the standard method for the identification of viral pathogens in CSF samples (35).

Currently, the commercially available BioFire® FilmArray® Meningitis/Encephalitis (ME) Panel is an FDA-approved (2015) multiplex method that simultaneously detects 14 pathogens, including bacteria, viruses, and fungi, in CSF samples (18, 36, 124). However, several researchers have reported false-positive and false-negative results from ME panels, particularly for Cryptococcus, Human Herpes Simplex Virus 1 and 2 (HSV-1 & HSV-2), and this concern has been reported in previous studies (6, 35). In addition, it is not suitable for CSF samples obtained by indwelling CNS medical devices.

## 4 Molecular-based diagnostic platforms for diagnosing meningitis/encephalitis

### 4.1 Conventional PCR and multiplex PCR assays

Polymerase chain reaction (PCR) has emerged as a definitive and sensitive diagnostic tool for meningitis and encephalitis (20, 37). Conventional PCR assays detect and identify specific pathogens by amplifying target sequences of DNA or RNA in clinical samples (20, 38). Multiplex PCR was used to detect multiple pathogens simultaneously in a single test. These molecular diagnostic methods provide rapid and accurate results in the detection of pathogens associated with CNSI (39). Additionally, multiplex PCR has the advantage of being able to identify and determine a wide range of pathogens in a single test directly from CSF samples, which can be particularly beneficial in the case of CNSI, where the causative agents can be diverse, such as bacteria, fungi, viruses and parasites (37, 40). However, there are several limitations, including high costs, especially in developing countries where resources may be limited (39, 41). Other limitations include the need for specialized equipment, trained personnel to perform the tests, and chances of false negatives and false positives due to contamination or inadequate sample collection (34, 42).

A study designed an in-house tetraplex-PCR assay to detect three causative bacterial pathogens, *S. pneumoniae*, *H. influenzae,* and *N. meningitidis*, and reported an analytical sensitivity of 792.3 copies/ml when all three pathogens were present in the sample. Clinical validation of the assay was tested in 150 CSF samples, and the assay was compared with a commercial multiplex real-time PCR kit (39). Similarly, another conventional multiplex PCR method was designed for the simultaneous detection of four bacterial pathogens and was validated in 447 CSF samples. A limit of detection of one pg/ml, positive percent agreement (PPA) of 100% (27/27), and negative percent agreement (NPA) of 96.7% (407/420) were reported compared with the culture method as the reference standard (28). Another study developed multiplex PCR coupled with capillary electrophoresis for the simultaneous detection of 18 pathogens. The performance of the assay was validated in 127 CSF samples from children suspected of having viral encephalitis. The concurrence rate between the multiplex PCR and sequencing assays was 91.5%. However, clinical validation was performed in a small sample size and only in patients with viral encephalitis. Further multiplex PCR assays were performed using leftover CSF samples submitted for culture (43). In addition, a prospective study validated PCR in 515 pediatric CSF samples and reported more rapid and sensitive methods than culture methods for the detection of infectious agents (44). Another prospective study from India designed and evaluated conventional multiplex PCR for the simultaneous detection of six bacterial pathogens. The assay was validated in 113 CSF samples from meningitis patients of all age groups, the overall detection rate of the assay was 6%, and the detection limit of the assay was one pg/µl. However, among the samples tested for clinical validation, only a few samples were known to be positive for CNSI (41). In one study, laboratory-developed and commercial PCR-based panels for the identification of bacterial pathogens of CNSI were compared by testing different gene targets, such as *ctrA*, *crgA*, and *nspA* for *N. meningitidis*, *ply* for *S. pneumoniae*, *P6*, and *bexA* for *H. influenzae.* The assay was evaluated in 110 bacterial strain isolates from 134 clinical samples, and the results revealed that in-house PCR is more sensitive, specific, and rapid than commercial PCR-based methods are. Genes such as *ply*, *ctrA*, *bexA*, *prf*, *meth*, and *cyl* have greater detection rates for identifying six bacterial pathogens, including *S. pneumoniae*, *N. meningitidis*, *H. influenzae*, *L. monocytogens*, *E. coli* and *S. agalactiae,* and the validation was tested in 100 CSF samples (45). Similarly, a cross-sectional study using 196 clinical CSF samples obtained from patients with suspected bacterial meningitis revealed that PCR was more sensitive and rapid because the gene targets *cspB* for *S. agalactiae*, *ctrA* for *N. meningitidis*, *ply*, and *lytA* for *S. pneumoniae*, and *bex* for *H. influenzae type b* (46). A study performed multiplex PCR to evaluate the diagnostic yield in 106 cases enrolled for the diagnosis of purulent meningitis in children reported a sensitivity of 100%, 88.9%, and 75% for *S. agalactiae*, *S. pneumoniae*, and *E. coli K1*, respectively, with a specificity greater than 91.3% for all three bacteria compared with culture as the reference standard (47). A study from India developed an in-house 16S rDNA-based nested PCR for the detection of eight bacterial pathogens, and validation in 150 CSF samples showed a sensitivity and specificity greater than 92% (48).

A newly developed duplex PCR method identifies *Mycobacterium tuberculosis* IS6110 and the eubacteria 16S rDNA sequence and simultaneously identifies tuberculous meningitis and bacterial meningitis in a single reaction. The duplex assay was tested in 150 clinical CSF samples for both tuberculous meningitis and bacterial meningitis and showed an overall specificity of 96.5% and an analytical sensitivity (limit of detection) of 103 cfu/ml for eubacteria and 102 cfu/ml for *M. tuberculosis*. However, this assay does not include an internal control (49). In addition, a novel single-tube nested PCR-lateral flow strip test (STNPCR-LFST), which targets the IS6110 gene, was designed to detect *M. tuberculosis*. This method was evaluated in CSF samples and reported 89% sensitivity and 100% specificity for detecting tuberculous meningitis, with better sensitivity than traditional PCR and a detection limit of one fg for tubercle bacilli DNA. Furthermore, it is a rapid and simple method with a sensitivity equivalent to that of the conventional two-step nested PCR method. In addition, STNPCR-LFST reduces the chances of cross-contamination and turnaround time (50). A prospective study evaluated the potential role of IS6110 and protein antigen b detection by PCR in the diagnosis of tuberculous meningitis in children. Clinical validation in 55 tuberculous meningitis cases and 20 controls revealed that PCR-based detection of both IS6110 and protein antigen *b* in CSF samples has greater sensitivity and 100% specificity than smear, LJ, and BACTEC cultures do (51). Another study using nested PCR targeting p32, IS6110, mtp40, and internal fragment of mtp40 gene fragments for tuberculous meningitis patients revealed 98% sensitivity and 92% specificity (52) (Table 1). Several previous studies used traditional two-step nested PCR-based detection of the IS6110 sequence for the detection of tuberculous meningitis and reported high sensitivities ranging from 75% to 98% (50). Many multiplex PCR assays have been developed in previous studies. These multiplex PCR assays are more effective than conventional culture methods in detecting pathogens causing meningitis. (Table 1) describes the various gene targets used, and the sensitivity and specificity of the molecular assays used to identify the etiology of CNSI.

**Table 1:** Gene targets used in conventional PCR, Multiplex PCR, Real-time PCR, LAMP, laboratory developed tests and commercial assay-based detection of meningitis/encephalitis in CSF sample.

Pathogens	Genes targeted	Platform/test	Sensitivity (%)	Specificity (%)	References
*Streptococcus pneumoniae*	*lytA, ply, plyA, gyrB*	Multiplex Light mix real-time PCR Multiplex real-time PCR Multiplex PCR qPCR-HRM Multiplex PCR Luminex	NA	NA	(4, 59, 66, 72, 111–115)
	*lytA*	Real time PCR	100	95.5/100	(38)
	*piaB*		95.3	99.5	
	*GntR-familySP2020*		100	99.8	
	*ply*		100	81	
	*lytA*	LAMP	100	100	(38)
	*SPNA45_01710*		95.7	100	
	*lytA-1*	Multiplex PCR	NA	NA	(43)
	*lytA*	Tetraplex PCR	92.8	95.1	(39)
	*lytA*	Real-time PCR	>95	>95	(60)
	*lytA*	Multiplex conventional PCR	>95	>95	(28)
	*lytA*	Multiplex qPCR	>90	>90	(67)
	*lytA*	Real-time PCR	NA	NA	(54)
	*lytA*	Real-time PCR	NA	NA	(65)
	*lytA*	Seeplex Meningitis ACE detection kit/multiplex PCR	NA	NA	(116)
	*lytA*	Multiplex PCR/Hybridization			(22)
*Haemophilus influenzae*	*Hpd, bexA, P6*	Multiplex Light mix real-time PCR Multiplex real-time PCR Multiplex PCR qPCR-HRM	NA	NA	(4, 59, 66, 111, 113–115, 117)
	*bexA*	Real-time PCR	100	90–100	(38)
	*omP2*		97.1	100	
	*omP6*		NA	NA	
	*licA*		97.1	99.1	
	*hpd*		95/95.7	91/92.3	
	*fucK*		97.1	100	
	Hibcapsule	LAMP	100	100	(38)
	*pstA*		80	100	
	*hpd*	Real-time PCR	NA	NA	(65)
	*fucK*	Multiplex PCR	NA	NA	(43)
	*Lic*	Multiplex PCR/Hybridization	NA	NA	(22)
	*hpd*	Tetraplex PCR	92.8	95.1	(39)
	*P6*	Multiplex conventional PCR	>95	>95	(28)
	*MOMP*	Real-time PCR	NA	NA	(54)
	*P6*	Seeplex Meningitis ACE detection kit/multiplex PCR	NA	NA	(116)
*Neisseria meningitidis*	*ctrA, nspA*	Multiplex Light mix real-time PCR Multiplex real-time PCR multiplex PCR qPCR-HRM	NA	NA	(4, 17, 59, 66, 111, 113–115)
	*ctrA*	Real-time PCR	76.1	NA	(38)
	*sodC*		99.6/94.7	100/77.9	
	*crgA*		93	96	
	*porA*		96.1	91.6	
	*ctrA*	LAMP	89/100	100/98.9	(38)
	*NMO_1242*		100	100	
	*CtrA*	Multiplex PCR	NA	NA	(43)
	*sodC*	Real-time PCR	NA	NA	(65, 66)
	*sodC*	Tetraplex PCR	92.8	95.1	(39)
	*ctrA*	Multiplex qPCR	>90	>90	(67)
	*ctrA*	Multiplex conventional PCR	>95	>95	(28)
	*CtrA*	Real-time PCR	NA	NA	(54)
	*crgA*	Real-time PCR	>95	>95	(60)
	*ctrA*	Seeplex Meningitis ACE detection kit/multiplex PCR	NA	NA	(116)
	*OpaA*	Multiplex PCR/Hybridization	NA	NA	(22)
*Streptococcus agalactiae*	*cfb*	Multiplex Light mix real-time PCR Multiplex PCR	NA	NA	(4, 113, 117)
	*Sip*	Multiplex PCR	NA	NA	(43)
	*dltS*	Real-time PCR	96.1	95.9	(38)
	*cfb*	Multiplex real-time PCR	>90	>90	(67)
	*fbsA*	Multiplex conventional PCR	>95	>95	(28)
	*cfb*	Real-time PCR	NA	NA	(54, 114)
	*Cfb*	Seeplex Meningitis ACE detection kit/multiplex PCR	NA	NA	(116)
*Listeria monocytogenes*	*hlyA, Hly*	Multiplex Light mix real-time PCR Multiplex PCR	NA	NA	(4, 117)
	*hly*	Multiplex PCR	NA	NA	(43, 113)
	*Iap, hly*	Real-time PCR	NA	NA	(54, 114)
	*Hly*	Seeplex Meningitis ACE detection kit/multiplex PCR	NA	NA	(116)
*Mycobacterium tuberculosis*	*IS6110, MPB64* and *HspX*	LAMP	43–88	80–100	(34)
	*IS6110, p32, mtp40,* internal fragment of *mtp40*	Nested PCR	>90	>90	(52)
	*IS6110*	Multiplex PCR	NA	NA	(43)
	*IS6110*	Real-time PCR	NA	NA	(13, 114)
	*MPB64*	Multiplex PCR/Hybridization	NA	NA	(22)
	*IS986*	Multiplex PCR Luminex	>90	>90	(112)
	*rpoB*	Xpert MTB/RIF	27–85	94.8–100	(34)
	*rpoB, IS6110, IS1081*	Xpert MTB/RIF Ultra	44.19–92.9	93.9–100	(34)
	Standard sequences in the database	mNGS	44–88.9	86.7–100	(34)
*Candida albicans*	*26s*	Real-time PCR	NA	NA	(114)
*Cryptococcus neoformans*	*URA*	Multiplex PCR Luminex	>90	>90	(112)
	*CAP*	Multiplex PCR	NA	NA	(43)
	*18S rDNA, 5.8S& ITS*	Real-time PCR	NA	NA	(54, 114)
	*18S rRNA*	Multiplex PCR/Hybridization	NA	NA	(22)
*Aspergillus spps*	*5.8s &ITS*	Real-time PCR	NA	NA	(114)
Varicella Zoster Virus	*ORF66*	Seeplex Meningitis ACE detection kit/multiplex PCR	NA	NA	(116)
	• *ORF29* • DNA Polymerase	Multiplex PCR/Hybridization	NA	NA	(22)
	*ORF21*	Multiplex PCR	NA	NA	(43)
Herpes Simplex Virus 1&2	*Gene 42, TK*	Multiplex PCR Luminex	80–100	>90	(112)
	*UL52*	Multiplex PCR	NA	NA	(43)
	• Glycoprotein D • Untranslated region44 • DNA polymerase	Multiplex PCR/Hybridization	NA	NA	(22)
	*UL29*	Real-time PCR	NA	NA	(13)
	*UL30, gD*	Seeplex Meningitis ACE detection kit/multiplex PCR	NA	NA	(116)
Cytomegalovirus	*UL54*	Seeplex Meningitis ACE detection kit/multiplex PCR	NA	NA	(116)
	*UL54*	Multiplex PCR	NA	NA	(43)
	• Glycoprotein O • Untranslated region 83 • Morphological transformation region II	Multiplex PCR/Hybridization	NA	NA	(22)
Epstein Barr Virus	*EBNA1*	Seeplex Meningitis ACE detection kit/multiplex PCR	NA	NA	(116)
	Repeat region 3–9	Multiplex PCR	NA	NA	(43)
Human Herpes Virus -6	*U38*	Seeplex Meningitis ACE detection kit/multiplex PCR	NA	NA	(116)
	*U4*	Multiplex PCR	NA	NA	(43)
	DNA Polymerase	Multiplex PCR/Hybridization	NA	NA	(22)
Human Enterovirus	*5*′-*UTR*	Seeplex Meningitis ACE detection kit/multiplex PCR	NA	NA	(116)
	*5*′*UTR*	Multiplex PCR	NA	NA	(43)

LAMP, loop-mediated isothermal amplification; mNGS, metagenomic next-generation sequencing; PCR, polymerase chain reaction; NA, not available.

### 4.2 End-point real-time PCR assays

Real-time PCR assays have emerged as valuable tools for identifying and detecting meningitis pathogens in CSF samples (20, 38, 53). These assays utilize primers and fluorescent probes to target and amplify the DNA or RNA of pathogens and provide rapid and sensitive detection of pathogens (54–56). Real-time PCR assays provide several advantages over conventional methods for the detection of pathogens of CNSI (6, 57). These methods include automation, increased sensitivity, and specificity and yield rapid results within 2 h after the DNA extraction process. Furthermore, real-time PCR assays can detect multiple pathogens simultaneously, enabling efficient and comprehensive testing (41, 58, 59). Additionally, the use of automated DNA extraction, liquid handling, PCR, and analysis on a single platform allows for high-throughput and quick turnaround times of clinical samples, leading to faster diagnosis and timely treatment (34, 60, 61). However, several drawbacks need to be considered. The utility of real-time PCR in low-resource settings is limited because of the high cost of the equipment and reagents involved; hence, conventional methods are still more feasible in low-resource settings (62, 63). Additionally, the requirement of technical expertise to perform the assay and interpret the results of real-time PCR assays may not be readily available in all healthcare facilities, which may lead to challenges for the utility of real-time PCR (32, 64).

Multiplex real-time PCR assays have been developed for the direct detection of three targeted bacterial pathogens, *S. pneumoniae*, *H. influenzae*, and *N. meningitidis,* in clinical samples without the need for DNA extraction (53, 65, 127). Similarly, another study designed a multiplex real-time PCR panel for the detection of six pathogens in CSF samples. This method was evaluated in 296 CSF samples from hospitalized patients with suspected bacterial meningitis. Among the 296 samples, 45 patients were suspected of having bacterial meningitis. Among the 45 patients with suspected bacterial meningitis, 32 CSF samples (70%) tested positive by microscopy, culture methods, molecular methods, and latex agglutination tests. However, only 15 CSF samples (approximately 33%) tested positive for meningitis by real-time PCR. The remaining 251 CSF samples were negative according to all the tested methods. The limitation of the assay was that the validation was performed with a relatively small sample size (54). A recent study from Mexico evaluated the role of real-time PCR in CSF samples as a diagnostic test for bacterial meningitis. A total of 512 CSF samples were obtained from suspected cases of CNSI and tested via real-time PCR for three pathogens, *S. pneumoniae, N. meningitidis*, and *H. influenzae,* which showed a diagnostic sensitivity and specificity of 100% and 95.46%, respectively, compared with culture methods (20). One study developed a duplex real-time PCR assay using SYBR Green integrated with melt curve analysis to detect *S. pneumoniae* and *N. meningitidis* in CSF samples, which was evaluated in 132 CSF samples from suspected bacterial meningitis patients and reported to have 100% sensitivity and specificity. However, a limitation of this study was that internal controls were not included, and a few CSF samples were positive for *S. pneumoniae* (22/132) and *N. meningitidis* (18/132). Therefore, this assay requires further validation in a larger sample size (60). Similarly, another study developed an Eva Green dye-based real-time PCR method for the detection of *S. pneumoniae* and *N. meningitidis* and validated it in 53 positive and seven negative CSF samples, which showed 100% analytical specificity and sensitivity (10).

Multiplex PCR was developed to detect three bacterial pathogens, was validated in 122 CSF samples, and reported a sensitivity of 100% (66). In contrast, one study reported a diagnostic sensitivity and specificity of 100% and 92.9%, respectively, for multiplex PCR of 212 CSF samples tested to identify and detect bacterial meningitis pathogens (67) (Table 1). A study developed two multiplex real-time PCR assays to detect three bacteria (*S. pneumoniae*, *N. meningitidis*, *and H. influenzae*) and three viruses (Enteroviruses, Mumps virus, Herpes Simplex Virus). Among 292 CSF samples, bacterial DNA was detected in 12 (4.1%) samples, and viral nucleic acid was detected in 94 (32%) samples. Compared with culture methods, bacterial real-time PCR showed an overall diagnostic sensitivity and specificity of 100% and 97.2%, respectively (55). A previous study developed a fluorescent hydrolysis probe-based TaqMan real-time PCR for the simultaneous detection of bacteria and fungi (11 gram-positive bacteria, nine gram-negative bacteria, and nine fungal species) in CSF samples. The assay was tested in 137 CSF samples and was shown to have a greater positive rate than the culture method. However, the limitation of this assay is that the conserved sequence selected is short region, and the designed universal primers and probes are not optimal, which may affect the sensitivity and specificity of the assay (68).

In an Indian study, rpoB, IS6110, and MPB64 were used as gene targets to assess the clinical utility of real-time PCR for the detection of tuberculous meningitis in 110 patients. Drug resistance was detected via high-resolution melt curve (HRM) analysis via rpoB gene amplicons, and the study reported 83.63% sensitivity and 100% specificity. Additionally, rpoB and MPB64 real-time PCR exhibited greater sensitivity than IS6110 real-time PCR (76.36%) in detecting rifampicin resistance in three out of 110 patients with tuberculous meningitis (69). In contrast, a previous study developed real-time PCR for the detection of tuberculous meningitis and compared its diagnostic performance with that of GeneXpert (Xpert), Mycobacteria Growth Indicator Tube (MGIT), and multiplex PCR (MPCR). The optimal cycle threshold (Ct) of real-time PCR was determined by comparing different gene targets of tubercle bacilli (*IS6110*, *16SrRNA*, *HSP65*, and *Ag85B*) and reported sensitivities of 36.7%, 21.1%, 16.7%, and 6.7%, respectively, and specificities of 97.6%, 100%, 100%, and 100%, respectively, for the IS6110RT‒PCR, MPCR, Xpert, and MGIT detection methods against the composite reference standards of definite, probable, and possible tuberculous meningitis; the sensitivity of the IS6110RT‒PCR method was greater than those of the 16SrRNA, HSP65, and Ag85B methods (63). The diagnostic performance of GeneXpert and multiplex PCR was tested using IS6110, MPB64, and protein B genes in 225 CSF samples to diagnose tuberculous meningitis, and MPCR was found to be a more sensitive method than GeneXpert (70). Moreover, several other investigations have shown that, compared with traditional PCR, IS6110 gene-based real-time PCR has an average sensitivity of 86% for diagnosing tuberculous meningitis (58, 71, 72).

Currently, commercially available panels, such as the Biofire FilmArray® ME Panel (BioFire, bioMerieux, Salt Lake City, USA), are used for the evaluation of CSF samples. A prospective study evaluated the ME panel in 1,560 CSF samples and reported that the sensitivity of the ME panel ranged from 85.7% for HHV-6 to 100% for nine of the 14 pathogens. The specificity of the ME panel was 99.2% or greater for all 14 pathogens. Additionally, according to the results from the meta-analysis of the ME panel, the pooled sensitivity was 90.2%, and the sensitivity was 97.7%. However, investigators have raised concerns about using ME panels because of false negatives and false positive results (3, 9, 73, 74) (Table 2). In a multicenter study, a multiplex qualitative QIAstat-Dx Meningitis/Encephalitis (ME) Panel was compared with a Biofire Film array panel, which included 585 retrospective residual CSF samples and analyzed 367 surviving samples. Compared with the BioFire FilmArray ME Panel, the QIAstat-Dx ME Panel had 100% positive percent agreement (PPA) for *Neisseria meningitidis*, *Streptococcus agalactiae*, *Escherichia coli* K1, *Listeria monocytogenes*, and Cryptococcus species in clinical samples. The negative percent agreement (NPA) value of the QIAstat-Dx ME Panel was >99% for each of the six bacterial species and one fungal target (21).

**Table 2:** Description of commercial or investigational molecular tests available for diagnosis of meningitis/encephalitis etiology and their features.

Test	Principle/platform	Targets detected/number of pathogens detected	Sample type	Sensitivity/specificity	Instrument	Processing time to results	Limitations	References
FilmArray® Meningitis/Encephalitis Panel (ME)	Qualitative multiplex PCR	14 pathogens bacteria/virus/fungi	CSF	>90%	Biofire Film array system	∼1–2 h	• Expensive • False positive and false negative results • *Mycobacterium tuberculosis* (TBM) not included in panel • Not suitable for CSF collected using CNS indwelling medical devices.	(1, 3, 18)
QIAstat- Dx meningitis/en cephalitis panel	Qualitative multiplex PCR cassette-based method	15 pathogens bacteria/virus/fungi	CSF	NA	QIA Stat-Dx analyzer system	<2 h	NA	(21)
POC SPCR QIAstat- Dx meningitis/encephalitis panel (QS)	Semi-quantitative multiplex PCR	15 pathogens bacteria/virus/fungi	CSF	NA	QIAstat-Dx analyzer system	∼1 h	NA	(118)
Syndromic Evaluation System (SES)	Multiplex nucleic acid amplification	22 pathogens bacteria/virus/fungi/parasite	CSF	>95%	SES System	7 h	• Expensive • Turnaround time ∼7 h • Requires dedicated instrument	(22)
Gene Xpert EV assay	Multiplex PCR	Enteroviral meningitis pathogens	CSF	>95%	GeneXpert system (Cepheid, Sunnyvale, CA, USA)	<2.5 h	Assay is limited for detection of only viral pathogens	(119)
Allplex™ Meningitis panel	Multiplex one-step real-time PCR	12 viruses, 6 bacteria	CSF	NA	Real-time PCR	∼2 h	• Labor intensive • Limited diagnostic performance	(120, 121)
Seeplex® Panel	Conventional PCR	12 pathogens bacteria/viruses	CSF	NA	DPO™ Technology	NA	Limited to bacterial and viral pathogens	(116)
FTD Bacterial/viral meningitis	Real-time PCR	6 viral and 3 bacterial targets	CSF, Blood	NA	Real-time PCR	∼90 min	• Labor intensive • Limited diagnostic performance	(121)
FTlyo Bacterial/Viral meningitis	Real-time PCR	6 viral and 3 bacterial Targets	CSF, Blood	NA	Real-time PCR	∼90 min	• Labor intensive • Limited diagnostic performance	(121)
VIASURE *H. influenzae* + *N. meningitidi*s + *S. pneumoniae* Real Time PCR Detection Kit	Real-time PCR	3 bacterial targets	CSF, Blood	NA	Real-time PCR	∼2 h	• Labor intensive • Requires electrically powered equipment for testing	(121)
NHS Meningitidis Real-TM	Real-time PCR	3 bacterial targets	CSF, Blood	NA	Real-time PCR	1 h	• Labor intensive • Limited diagnostic performance	(121)
Eazyplex CSF direct panel	LAMP	6 bacterial pathogens	CSF	>90%	Amplex Biosystems (GmbH, Giessen, Germany)	<1 h	Lack of data on clinical validation	(84)
FTD Neuro 9 panel	Reverse transcriptase multiplex real-time PCR	10 viral target pathogens	CSF, Blood	NA	Real-time PCR	∼90 min	• Labor intensive • Limited diagnostic performance	(121)
Meningitis Viral 1&2 ELITe MGB®Panel	Triplex PCR (for panel 1) and one step, reverse transcription, and real-time multiplex PCR (for panel 2)	Viruses	CSF	>95%	ELITe InGenius®	<1 h	NA	(122)
Multiplex Luminex assay	Multiplex PCR	5 pathogens (Bacteria/virus/fungi)	CSF	>90%	Multiplex PCR-Luminex system	NA	• False positive results • Limited diagnostic performance	(112)
Multiplex Light Mix-Real-time PCR	Multiplex real-time PCR	5 bacterial Pathogens	CSF	NA	Light Cycler 480-II (Roche)	<4 h	NA	(4)
Internally controlled multiplex TEC-LAMP assay	Real-time multiplex LAMP Technology	3 bacterial pathogens	CSF	>90%	Light Cycler® 480 instrument II (Roche Diagnostics, Sussex, UK)	NA	Requires Thymine residue for the assay	(91)
Advanced fragment analysis-based assay (AFA)	Quantitative, multiplexed gene expression analysis	22 pathogens Bacteria/virus/fungi	CSF	Sensitivity 63–100%, Specificity 98.2%	ABI Verity 96 Thermal Cycler	∼4–6 h	Requires separate equipment for fragment analysis and separation	(2)
NAATs	Specific PCR	Bacteria/virus/fungi	CSF	>90%	Thermocycler	1–6 h	Expensive	(6)
16SrRNA PCR	PCR detection of 16s ribosomal RNA	Bacteria/virus	CSF	NA	Thermocycler	Hours-days	Expensive and requires expertised personnel	(6)
PCR	Traditional PCR	Bacteria/virus/fungi	CSF	NA	Thermocycler	Hours	• Expensive • Requires lab expertise • Poor sensitivity	(6)
LAMP	Thermostatic, nucleic acid amplification and detection by color change	MTB	CSF	Sensitivity-43–88% Specficity-80–100%	NA	1 h	• No data on performance of assay • Variable sensitivity and specificity • CSF specimens require further evaluation	(6, 34)
Gene Xpert	Real-time fluorescence quantitative PCR, Cartridge-based PCR	MTB	CSF	Sensitivity-27–85% Specificity-94.8–100%	GeneXpert system	2.5 h	• Expensive • Poor sensitivity	(6, 34)
GeneXpert Ultra	High resolution melting, Cartridge-based PCR	MTB	CSF	Sensitivity (44–93%) Specificity (94–100%)	GeneXpert ultra system	<2 h	• Expensive • Variable sensitivity • Cannot rule out Tuberculous meningitis	(6, 30)
mNGS	Nucleic acid amplification, gene sequencing	MTB/etiology	CSF	Sensitivity (62%) Specificity (99%)	Sequencing platform	24–48 h	• Expensive • Chances of cross contamination	(6, 30)
Lab developed tests	Conventional PCR, one step-PCR, Duplex, Tetra-plex Multiplex real-time PCR	Bacteria	CSF	>90%	PCR and Real-time PCR	NA	Variable sensitivity and specificity	(39, 54, 60, 67, 123)
Purulent meningitis-TaqMan Aarry Card (PM-TAC)	Multiplex real-time PCR, Microfluidics high throughput technology	16 bacteria and 5 fungi	CSF	>95%	ViiA7 real-time PCR system (LifeTechnologies)	3 h	Expensive for lower resource settings	(114)
CNS-TAC assay	Microfluidic technology	6 bacterial pathogens, 13 viruses and 2 parasites	CSF	85.6% Sensitivity, 96.7% Specificity	ViiA7 real-time PCR (Applied Biosystems, Foster City, CA)	2.5 h	• Requires dedicated instrument • Fungal pathogen such as Cryptococcus neoformans not included in the panel	(78)

CSF, Cerebrospinal fluid; CNS, Central nervous system; LAMP, Loop mediated isothermal amplification; mNGS, Metagenomic next generation sequencing; MTB, *Mycobacterium tuberculosis*; NAATs, Nucleic acid amplification tests; PCR, Polymerase chain reaction; TBM, Tuberculous meningitis; NA, Not available.

A retrospective study compared the diagnostic utility of a commercially available Multiplex Syndromic Evaluation System (SES), which was tested in 110 patients, with that of the standard culture method and reported that SES has a sensitivity of 42.18% and specificity of 100%. However, this study has the limitation of a small sample size. Furthermore, other commercial molecular panels that detect bacterial, fungal, or viral pathogens are available, but clinical validation of these panels is still lacking (Table 2). Therefore, further investigations are needed to test the clinical significance of the SES (75). Advanced cartridge-based real-time PCRs, such as GeneXpert MTB/RIF and GeneXpert MTB/RIF ultra, are commercially available and are fully automated systems designed by Cepheid (United States) for the simultaneous identification of tubercle bacilli and rifampicin resistance in CSF and other clinical specimens. Various studies have revealed that the diagnostic performance of GeneXpert MTB/RIF is suboptimal for tuberculous meningitis (76) and has a combined sensitivity of 24% to 86% and specificity of 98.6% (70, 77). Although the GeneXpert MTB/GeneXpert MTB RIF Ultra has high specificity, it lacks promising sensitivity and has a negative predictive value for ruling out tuberculous meningitis. However, GeneXpert Ultra is transformational and has more benefits than Xpert in the diagnosis of tuberculous meningitis. A commercial TaqMan array card (CNS-TAC) was recently designed that detects twenty-one pathogens simultaneously, and its clinical validation in 120 CSF samples showed 85.6% sensitivity and 96.7% specificity (78). Tables 2, 3 summarize the sensitivity, specificity, performance, limitations, and benefits of these methods.

**Table 3:** The diagnostic performance of selected tests for the diagnosis of etiology of meningitis/encephalitis in CSF sample.

Diagnostic test	Limit of detection (CFU/ml)/particles/ml	Sensitivity (%)	Specificity (%)	Role in current practice	Benefits	References
Film Array® Meningitis/Encephalitis Panel (ME)	NA	>90	>90	• Widely used • First FDA approved panel for ME diagnosis	• Rapid • Identify up to 14 targeted pathogens	(3)
QIAstat- Dx meningitis/encephalitis panel	NA	NA	NA	CE-IVDD marked device	• Identify up to 15 targeted pathogens • Performance similar to ME panel	(21, 31)
Syndromic Evaluation System (SES)	• 0.1–50 viral particles/ml of CSF • 100–200 bacterial cells/ml of CSF • 5 parasites/ml of CSF	>95	>95	NA	• Simultaneous detection of multiple pathogens • High specificity	(22)
LAMP	NA	43–88	80–100	Conditional recommendation	• Rapid and inexpensive • No special equipment is needed • Results are easy to interpret	(34)
Traditional NAATs	NA	68–82	90–100	Used in current practice	High specificity	(25)
GeneXpert	∼110–116 CFU/ml	27–85	94.8–100	WHO Recommended method (2015) for early diagnosis	• Rapid test • High specificity • MTB and RIF resistance detection • Semi-quantification of bacillary load	(34)
GeneXpert ultra	∼10–15.6 CFU/ml	44.19–92.9	93.9–100	WHO Recommended method (2017) for early diagnosis	Higher sensitivity than Gene Xpert	(34)
CRISPR-MTB	NA	73	98	NA	• Requires less volume of CSF sample • High specificity	(42)
mNGS	NA	44–88.9	86.7–100	Not recommended for early diagnosis	Broad pathogen coverage	(30, 34)

CRISPR-MTB, clustered regularly interspaced short palindromic repeats-*Mycobacterium tuberculosis*; CSF, Cerebrospinal fluid; LAMP, Loop-mediated isothermal amplification; mNGS, Metagenomic next generation sequencing; MTB, Mycobacterium tuberculosis; NAATs, Nucleic acid amplification tests; RIF, Rifampicin; NA, Not available.

### 4.3 Loop-mediated isothermal amplification-based assays

In recent years, loop-mediated isothermal amplification (LAMP) assays have gained attention as alternative methods for the detection of CNS pathogens (56, 79). LAMP assays were developed in 2000 and 2011 (125, 126) (Figure 1). LAMP amplifies specific DNA targets via two primer sets, buffers, and DNA polymerase, and its efficiency is not affected by PCR inhibitors (34, 58, 80). These assays offer several advantages over conventional PCR, including simplified and rapid amplification techniques that do not require complex thermocycling equipment (79, 80). LAMP assays are more sensitive and specific for detecting a broad range of CNS pathogens, including bacteria, fungi, and viruses. Furthermore, LAMP assays can be performed in point-of-care settings, allowing for rapid diagnosis and initiation of appropriate treatment (81–83). These assays hold great promise for the early and accurate diagnosis of CNSI, particularly in resource-limited settings where access to sophisticated laboratory equipment is limited (84).

Various LAMP assays have been developed for the diagnosis of bacterial pathogens (Table 1). A 16S rRNA-based one-tube LAMP assay was designed for the detection of *S. aureus*, *S. pneumoniae*, *S. suis*, and *S. agalactiae*. The results demonstrated that the LAMP assay was more sensitive and specific than conventional PCR and lacked cross-reactivity with other targeted pathogens, such as *H. influenzae, N. meningitidis*, and *Escherichia coli*. However, this assay was performed on cultured isolates but has not been validated in CSF samples; therefore, further testing in clinical samples is needed (85). Similarly, a real-time fluorescence LAMP assay was designed to detect *Streptococcus agalactiae* (62). A study on a LAMP assay for the detection of *N. meningitidis* reported 94% sensitivity and 100% specificity (83).

A recent study from India developed a multitargeted LAMP (MLAMP) assay using the gene targets *sdaA*, IS1081, and IS6110, which was validated in 300 CSF samples and showed a diagnostic sensitivity of 88% and specificity of 100% compared with probable cases of tuberculous meningitis and the conventional culture method (79). A meta-analysis of LAMP revealed a combined sensitivity of 77% and specificity of 99% for diagnosing extrapulmonary tuberculosis (86). In contrast, the IS6110-based LAMP assay provided 43% sensitivity and 92.9% specificity for diagnosing tuberculous meningitis (87). A study from India retrospectively evaluated the LAMP assay in 27 CSF samples and reported 88% sensitivity and 80% specificity in comparison with nested PCR as the reference standard, which produced 52.9% specificity and 90% specificity for detecting tuberculous meningitis (81). However, it is uncertain whether LAMP technology can be used as a rule-out test in TB diagnosis. Thus, further research is needed to demonstrate the effectiveness of LAMP in diagnosing tuberculous meningitis. Visible DNA microarray platforms and LAMP were designed to detect Cryptococcus species in CSF samples by targeting the ITS region and CAP59 gene. Among the 133 CSF samples tested, 73% were correctly identified by the *CAP59* gene, whereas 45.5% were correctly identified by the ITS region. The *CAP59* gene correctly identified 100% of the Cryptococcus isolates, and the ITS platform correctly detected 70% of the Cryptococcus isolates. However, for CSF samples, amplification was observed in only 55.5% of *C. neoformans* (88). In addition, the LAMP-based microfluidic model was designed for the detection of Cryptococcus (89). Furthermore, researchers from China have evaluated five methods (India ink, conventional culture, LAMP, LFA, and real-time PCR) to diagnose cryptococcal meningitis in non-HIV-infected patients; of the 85 CSF samples tested, the lateral flow assay (LFA) had a high positive detection rate of 97.6%, whereas LAMP had a positive rate of 87.1% compared with that of India ink, culture, and real-time PCR assays (90). Multiplex LAMP assays are difficult to design. However, a novel modified LAMP assay called *Tth* Endonuclease Cleavage (TEC) LAMP was developed. It is based on the *Tth* endonuclease IV with unique primers and probes to simultaneously detect bacterial meningitis pathogens such as *S. pneumoniae*, *N. meningitidis*, and *H. influenzae.* In this assay, 168 bacterial strains were tested, and the results demonstrated that the TEC-LAMP assay was 100% specific, with limits of detection for *S. pneumoniae, N. meningitidis*, and *H. influenzae* of 39.5, 17.3, and 25.9 genome copies per reaction, respectively. Additionally, 65 archived PCR-positive samples were tested for clinical performance, and the results revealed a diagnostic sensitivity and specificity of 92.3% and 100%, respectively (91). A commercial multiplex real-time LAMP-based Eazyplex® CSF direct M panel is available for detecting bacterial meningitis pathogens. The clinical validation data of the panel are still lacking (Table 2) (84). A paper-based device was developed in a study to perform LAMP with simultaneous real-time detection of several DNA targets. In three CSF DNA samples from confirmed CNSI cases, the developed platform detects up to 10^2^–10^5^ copies of genomic DNA, and the paper-based platforms are easy to use, economical, and suitable for point-of-care testing (92).

### 4.4 Next-generation sequencing and metagenomic next-generation sequencing assays

The use of metagenomic next-generation sequencing (mNGS) and next-generation sequencing (NGS) assays for the diagnosis of CNSI has gained popularity in recent years (93). These methods can simultaneously detect multiple pathogens, including viruses, bacteria, and fungi, in a single test and offer several advantages over traditional methods. Furthermore, NGS and mNGS have the potential to identify novel or rare pathogens that may not be targeted by traditional diagnostic methods (94). Lin et al*.* reported that NGS integrated with culture could improve the detection rate for diagnosing tuberculous meningitis (95). A retrospective analysis of CSF samples from 66 suspected tuberculous meningitis patients reported increased sensitivity (65.8%) and specificity (71.4%) of mNGS compared with traditional methods (96). In another study, an mNGS assay was designed and validated for the diagnosis of CNSI in 95 CSF samples, and the results revealed 73% sensitivity and 99% specificity compared with those of standard methods. Furthermore, the assay was tested in 20 known positive CSF samples and showed 92% sensitivity and 96% specificity compared with conventional methods (97). Similarly, another study tested 74 residual CSF samples by mNGS and reported that the diagnostic accuracy, sensitivity, and specificity of mNGS with respect to conventional methods were 100%, 95%, and 96%, respectively (94). A retrospective examination of CSF samples from 51 suspected tuberculous meningitis patients examined by mNGS in comparison with four other tests revealed that mNGS had a much higher diagnostic sensitivity than AFB (84.4%), MGIT 960 (22.2%), MTB PCR (24.4%), and Xpert (40%) (98). Another retrospective study revealed greater sensitivity (66.7%) of mNGS than other methods, including Z-N staining (33.3%), PCR (25%), and culture (8.33%) (99). However, the interpretation of mNGS along with modified Z-N staining or Xpert can improve the diagnostic sensitivity of tuberculous meningitis (14). A recent systematic review and meta-analysis of mNGS for diagnosing tuberculous meningitis revealed a pooled sensitivity of 62% and specificity of 99% (30). In the diagnosis of Cryptococcal meningitis in HIV-negative individuals, mNGS showed 93.5% sensitivity, 96% specificity, and 95.4% concordance. However, mNGS has less sensitivity and concordance than Crag tests do (97.4%) (26). Despite the moderate sensitivity of mNGS, it has high specificity for pathogen detection. However, its clinical significance remains unclear. Therefore, further research is needed to assess the role of mNGS in the diagnosis of CNSI. The diagnostic performance of common molecular diagnostic techniques is summarized in Table 3.

### 4.5 Other techniques for the diagnosis of meningitis/encephalitis

The high-resolution melting curve technique offers a promising approach for diagnosing CNSI. This technique uses the analysis of DNA melting curves to detect and differentiate specific pathogens responsible for these infections (2, 20, 100). It assesses the changes in DNA melting patterns and identifies the presence of bacterial, viral, or fungal DNA in CSF with high accuracy and specificity. This technique offers several advantages over traditional methods such as culture and PCR, including its quick turnaround time, cost-effectiveness due to probe-free detection, and potential to detect multiple pathogens simultaneously in a single test (19, 34, 101, 102). High-resolution melting (HRM) qualitative PCR was developed for the simultaneous detection of *N. meningitidis*, *H. influenzae*, and *S. pneumoniae* and is highly sensitive compared with TaqMan-based real-time PCR (59). The utility of HRM has been tested in 110 patients with tuberculous meningitis, and it was found to be effective in the detection of tuberculous meningitis and rifampin resistance (*rpoB*) (69). A polymer/paper hybrid microfluidic biochip integrated with LAMP that is instrument-free has been developed to detect *N. meningitidis*, *S. pneumoniae*, and *H. influenzae type b* (Hib), which is sensitive and specific and has the potential to be used for point-of-care diagnosis (103). The potential of the MALDI-TOF assay was assessed in smear-positive CSF samples, revealing that it is useful for quick identification of gram-negative rods directly in smear-positive CSF samples but not in gram-positive bacteria (104). An in-house-developed duplex recombinase polymerase amplification (RPA) assay was developed to detect *S. pneumoniae, N. meningitidis*, and *H. influenzae* and was compared with real-time PCR. The developed RPA was validated in 64 PCR-positive clinical samples and showed diagnostic sensitivities of 100%, 86.3%, and 100%, respectively, and 100% specificity for all three pathogens (105). Similarly, the real-time nucleic acid sequence-based amplification (NASBA) detection method is designed to detect RNA transcripts of *H. influenzae*, *N. meningitidis*, and *S. pneumoniae,* and validation of the assay showed 100% specificity (106). The diagnostic accuracy of digital PCR targeting the IS6110 gene for identifying tuberculous meningitis was tested in 101 patients, and the sensitivity of digital PCR (70.4%) was greater than that of Xpert MTB/RIF (29.6%) (32). The efficacy of the CRISPR-based assay for the treatment of tuberculous meningitis was tested in 27 CSF samples, and it reported higher sensitivity (73%) than did culture and Xpert (107). A previous multicentric study validated the commercially available Geno Type MTBDRplus line probe method for the detection of isoniazid- and rifampicin-resistant *Mycobacterium tuberculosis* isolates. The method was validated in 89 culture-positive *Mycobacterium tuberculosis* isolates and reported 93% sensitivity and 97% specificity for isoniazid resistance and 80% sensitivity and 98.8% specificity for rifampicin resistance. The results of the Geno Type MTBDRplus line probe method were compared with drug susceptibility testing reports of the BACTEC MGIT 960 system as the gold standard (108). In addition, Simple Label-free Imidoesters Microfluidica simple label-free imidoesters microfluidic system (SLIM) was developed and validated in 72 suspected cases of tuberculous meningitis in non-HIV patients. The results were compared with those of mycobacterial culture and GeneXpert as reference methods and revealed a sensitivity of 100% and a specificity of 92%. However, the SLIM method was tested in a small sample size, and further validation in larger samples is needed to confirm the utility of this assay (109). A novel multiplex PCR Mag-Array system was designed on the basis of chimeric primers, temperature switch PCR, and MagPlex-TAG techniques for the simultaneous detection of 13 viruses. This system was tested in 177 CSF samples and has high specificity (110). Overall, various studies have developed many molecular assays based on different methodologies. However, the results of these studies were limited in terms of sample size. Therefore, larger studies are needed to evaluate the clinical significance of these assays.

## 5 Conclusion

The clinical diagnosis of CNSI remains challenging due to the diverse etiologies of pathogens. Despite the existence of numerous diagnostic modalities, there are still drawbacks. For example, the use of Xpert Ultra for the detection of MTB, including mNGS, has low sensitivity. Currently available commercial platforms are expensive. However, significant advancements have been made in the development of molecular-based assays for the diagnosis of meningitis/encephalitis. The detection of host-based biomarkers in CSF, serum, or plasma such as microRNA levels and mixed signatures are yet to be studied in tuberculous meningitis, and pathogen-based biomarkers (example: RNA, volatile organic chemicals not yet explored in tuberculous meningitis) may aid in early diagnosis compared with nucleic acid testing. Furthermore, Rapid Diagnostic Tests (RDTs) and point-of-care tests provides rapid diagnosis that need to be strengthened. Emerging technologies such as the CRISPR-Cas9 technology and microfluidics-based systems have the potential to detect pathogens but require further investigation. Although cutting-edge technologies such as NGS and mNGS hold significant promise, due to extensive requirements for bioinformatics and sample processing, they are expensive. The role of NGS and mNGS remains unclear. Future research should focus on developing molecular techniques by improving the performance of existing targets and identifying alternative diagnostic targets to enhance sensitivity and specificity. Furthermore, rapid, cost-effective, and affordable tests are need to be developed for diagnosis in lower resource settings. However, all these molecular diagnostic tests cannot be used indiscriminately and need to be used in correlation with the clinical context. Also, it is important that any molecular diagnostic method implementation/utilization has to be done in partnership with the clinician. This ensures that there is clear understanding of the given test/method characteristics (sensitivity/specificity/false predictive values etc.) and interpretation of the result of molecular diagnostic tests with the clinical background and knowledge of the common local pathogens causing the CNSI improves the quality of the diagnosis.
